# Genetic Testing for Cancer Risk: Is the Community Willing to Pay for It?

**DOI:** 10.3390/ijerph18168752

**Published:** 2021-08-19

**Authors:** Azimatun Noor Aizuddin, Abdul Rahman Ramdzan, Sharifah Azween Syed Omar, Zuria Mahmud, Zarina A. Latiff, Salleh Amat, Keng Wee Teik, Ch’ng Gaik Siew, Haniza Rais, Syed Mohamed Aljunid

**Affiliations:** 1 Department of Community Health, Faculty of Medicine, Universiti Kebangsaan Malaysia, Jalan Yaacob Latif, Bandar Tun Razak, Cheras, Kuala Lumpur 56000, Malaysia; saljunid@gmail.com; 2 Department of Public Health Medicine, University of Malaysia Sabah, Jalan UMS, Kota Kinabalu 88400, Malaysia; 3 Faculty of Education, Universiti Kebangsaan Malaysia, Bangi 43600, Malaysia; shazween@ppukm.ukm.edu.my (S.A.S.O.); zuria@ukm.edu.my (Z.M.); sallehba@ukm.edu.my (S.A.); 4 Department of Paediatrics, Faculty of Medicine, Universiti Kebangsaan Malaysia, Jalan Yaacob Latif, Bandar Tun Razak, Cheras, Kuala Lumpur 56000, Malaysia; zarinaal@ppukm.ukm.edu.my; 5 Genetic Department, Hospital Kuala Lumpur, Jalan Pahang, Kuala Lumpur 50586, Malaysia; wtkeng@gmail.com (K.W.T.); chnggaiksiew@moh.gov.my (C.G.S.); 6 Department of Education Psychology & Counseling, International Islamic University Malaysia, Jalan Gombak, Selangor 53100, Malaysia; hanrais@iium.edu.my; 7 Department of Health Policy and Management, Kuwait University, 320 St, Hawally 13110, Kuwait

**Keywords:** willingness to pay, genetic testing, cancer, willingness to undergo, prevention

## Abstract

With the increasing number of cancer cases worldwide, genetic testing for familiar cancers seems inevitable, yet little is known on population interest and the monetary value for cancer genetic risk information. The current study aimed to determine the willingness to undergo and pay for cancer genetic testing among the Malaysian population. A self-administered questionnaire was distributed to cancer patients and their family members in the oncology and daycare units in several government hospitals. Of 641 respondents (354 patients, 287 family members), 267 (41.7%) were willing to undergo cancer genetic testing. The median that respondents were willing to pay was USD 48.31 (MYR 200.00) IQR USD 96.91 (MYR 400), while 143 (22.3%) respondents were willing to pay a shared cost with the insurance company. Regression analysis identified independent positive predictors of willingness to pay as respondent’s status as a family member, high education level, and willingness to undergo cancer genetic testing in general, while in patients, female gender and high level of education were identified as independent positive predictors. Generally, the population needs more information to undergo and pay for cancer genetic testing. This will increase the utilization of the services offered, and with cost-sharing practices with the provider, it can be implemented population-wide.

## 1. Introduction

One of the fastest evolving modalities for cancer screening available worldwide is genetic testing, which uses advanced genomics technology to revolutionize oncology care. An estimated 5% to 10% of cancers have a hereditary component [1]. Certain cancers such as breast, colorectal, ovarian, prostate, pancreatic, and endometrial cancers sometimes run in the family. Individuals with inherited mutations in known hereditary cancer genes can have a significantly increased chance of developing particular types of cancers. For example, Lynch syndrome, a hereditary colorectal cancer, is associated with mutations within the MLH1, PMS2, MSH2, MSH6, and EPCAM genes [2]. To detect such anomalies, predictive genetic testing is a powerful tool to recognize such individuals and significantly benefit both the patient and at-risk relatives [1]. Genetic testing should be focusing on patients with moderate and high risk [3]. The American Society of Clinical Oncology (ASCO) has affirmed that identifying and managing individuals with an inherited predisposition to cancer are core elements of oncology care [4]. More than 200 genetic tests are currently clinically available to determine the risk of developing various cancers [4]. If a person is found to have a gene variant, many things can be done to reduce the risk of developing cancer, such as making certain lifestyle changes, chemo-prophylaxis, and risk-reduction surgeries, thus leading to hopefully lower cancer death rates. Despite this, the literature suggests that predictive genetic testing is underutilized, with approximately half of at-risk individuals being tested [5].

Many studies generally found positive attitudes towards genetic testing for the risk of diseases including cancer [6]. Yet, not all individuals wish to pursue testing, and not all families with medical histories warrant testing [7]. Genetic testing raises unique issues related to the test results in terms of psychological, financial, and ethnocultural impacts for both the individuals and their families [8]. While the test information may reduce uncertainty, provide reassurance, assist in life-planning decisions, and benefit future treatment decisions, the limitations of genetic testing can be the reason for hesitancy of patients and families in deciding whether to be tested [9]. Selecting who should be the first in a family to obtain genetic testing adds a level of complexity. It is desirable to start testing with a family member who has had cancer, but that person may not be interested in being tested [10]. Thus, genetic counseling is recommended before and after the test to discuss the potential risks, benefits, and limitations of genetic testing [8] for cancer risk.

Willingness to pay (WTP) is a standard economic measure for health interventions that offer researchers the flexibility to investigate how people value a wide range of health benefits. WTP for a specified health improvement is defined as the maximum amount of money individuals could pay for a health improvement and still consider themselves better off [11]. It is used in cost–benefit analysis by estimating the intervention cost against the WTP values of the indicated improvement [12]. This kind of information helps to develop care plans that are more likely to be successful in terms of acceptability, as they incorporate patients’ preferences and may lead to more efficient use of resources [13]. Identifying the factors associated with WTP for genetic services is increasingly important, as testing is integrated into routine cancer care [14]. The high cost is a significant barrier to screening for multiple cancer types [15]. Cost-sharing practices may impact testing behaviors in high-risk individuals [14], yet the influence of out-of-pocket expenses on individual perceptions and actions in the setting of increased genetic cancer risk is less studied.

According to Malaysian Study on Cancer Survival (MySCan), out of 31.5 million in Malaysia, 43,837 new cancer cases were diagnosed in 2018 [16]. Despite this huge number of patients in the country, the concept of cancer and genetic testing is not familiar among Malaysians, except for those in the medical profession. There has been no survey in Malaysia with the explicit goal of exploring consumer interest in genetic tests. A previous study on breast cancer research in Malaysia [17] reported 82% of high-risk women accepted genetic counseling and genetic testing. Out of them, only 78% of gene carriers informed their families. However, only 11% of their relatives had come forward to take predictive genetic testing and genetic counseling even when it was offered free of cost. This low inclination for the real BRCA testing is supported by a study done in Poland with reasons of low knowledge, low motivation, and the high price of genetic testing [18].

These tests are generally offered to cancer patients and their relatives by healthcare providers working in specialized clinical settings but are restricted to some particular groups of patients or their relatives. More research is, therefore, needed regarding the interest of cancer genetic testing targeting the general population. This paper aims to gain insight into the readiness of cancer patients and their family members to undergo and share the cost for cancer genetic testing, generating important information to be integrated into cancer prevention programs.

## 2. Materials and Methods

A cross-sectional study was conducted at three tertiary care hospitals in Malaysia, namely Hospital Kuala Lumpur (HKL), the main reference hospital in Malaysia; Hospital Pulau Pinang (HPP), the cancer reference hospital for the north area of Malaysia; and the National Cancer Institute (IKN), from September 2018 until July 2019. The research population comprised consecutive cancer outpatients and the immediate person who accompanied them to the oncology clinic and daycare unit at these hospitals. The sample size was calculated prior to data collection accordingly. Out of a total of 680 individuals approached which were patients and their relatives, 641 agreed to participate, for a response rate of 94.2%. Those who were unfit to participate or did not provide consent were excluded from the study. The data were collected using a self-administered guided questionnaire, which was self-developed by the research team. The experts checked the content validity, and a pilot study was done to check its construct validity. Socio-demographic information included gender, race, religion, marital status, number of children, educational status, monthly income, and health insurance status. Personal history of cancer and the record of family history of cancer amongst first-degree and extended relatives were documented.

The study is a part of a larger course. Only the willingness-to-undergo and the willingness-to-pay components of the survey are discussed in this paper. Despite considerable methodological research into using different willingness-to-pay techniques, no consensus has emerged concerning the best practice, and different methods likely suit different situations and patient groups [19]. The simplest way of value elicitation is to use an ‘open-ended’ approach where the respondent is asked to provide a value based on his/her idea. A more sophisticated method is a ‘payment scale’ approach where the respondent chooses from the list of possible values provided [13]. Consumer surveys have a general limitation that an expression of interest does not necessarily represent uptake rate—a fact that has been discussed in many previous studies where actual uptake was much lower than anticipated [20]. Our survey asked the respondent’s willingness to both undergo and pay for cancer genetic testing to address the limitation. Price options were presented to respondents for out-of-pocket payment for cancer genetic tests so that participants could think in concrete terms of their out-of-pocket expenditure.

The questionnaire and study protocols were reviewed and approved by the Ministry of Health and the University Ethics Board before administration to the public. Respondents were informed that their participation was voluntary, and their responses would be kept confidential. Subjects were asked about their interest in undergoing cancer genetic testing if they are partially or fully funded by the government, share payment with insurance, or pay out-of-pocket. The respondents were also requested to state the amount they were willing to pay out-of-pocket for this testing. This range of questions probed the respondents’ reactions to the cost-sharing practice for these genetic test services and investigated the utility they attach to genetic risk information by asking how much they would pay. A combination of two elicitation methods was used in this study: open-ended question followed by one best answer for a pay scale ranging from USD 120.89 (MYR 500) to USD 725.34 (MYR 3000), with the aim that these values would provide a strength of preference that is not detectable using only the open-ended approach. WTP for cancer genetic test was positively skewed; hence, median WTPs are reported here. Descriptive and bivariate analyses were carried out to enlist the sample’s characteristics and assess if any statistical associations existed between the outcome measures and individual factors believed to impact willingness to undergo and WTP for this testing. The data were analyzed statistically using the Statistical Package for Social Sciences version 23.0 (IBM, Armonk, NY, USA).

## 3. Results

### 3.1. Participants’ Demographic Information

A total of 641 respondents completed the questionnaires. More than half of the respondents were cancer patients (354, 55.2%). Respondents were from three hospitals: 396 (61.8%) from Hospital Pulau Pinang (HPP), 130 (20.3%) from Institut Kanser Negara (IKN), and 115 (17.9%) from Hospital Kuala Lumpur (HKL). Three hundred and five (47.6%) respondents were between 40 and 60 years old, with a median age of 51 (IQR 22.5). The majority of the respondents were females (436, 68.0%), Malays (450, 70.2%), Muslims (453, 70.7%), and married (514, 80.2%) with more than one child. Among the respondents, 201 (31.4%) were university graduates. The respondents’ monthly income ranged from USD 48.35 (MYR 200) to USD 4835.72 (MYR 20,000), with a median income of USD 483.57 (MYR 2000) Interquartile range (IQR) USD 436.10 (MYR 1800). In addition, 529 (82.5%) respondents earned less than USD 1208.92 (MYR 5000). Only 146 (22.8%) respondents had private health insurance, and 340 (53.0%) respondents had a family history of cancer.

Only 267 (41.7%) respondents reported that they were willing to undergo genetic testing for cancer risk. Table 1 shows the WTP of respondents for cancer genetic testing. Only 98 respondents (15.3%) were willing to pay out-of-pocket, with a median of USD 48.35 (MYR 200) IQR USD 96.91 (MYR 400). Only 143 (22.3%) respondents were WTP if the total cost was shared with the insurance provider using a co-payment service. However, if subsidized or paid by the government, 291 (45.4%) respondents were willing to undergo genetic testing. If they were given full coverage from the insurance, 265 (41.3%) respondents were ready to undergo genetic testing.

### 3.2. Factors Associated with the WTP for Genetic Testing for Cancer Predisposition

Table 2 shows that there was a significant association between WTP and several socio-demographic factors. Percentages of WTP were higher among respondents who were aged less than 40 years old (*p* = 0.032), were females (*p* = 0.001), had no family history of cancer (*p* = 0.004), and had a high level of education (*p* < 0.001), with a monthly household income of more than USD 1208.88 (MYR 5000) (*p* < 0.001), private insurance (*p* = 0.011), and willingness to undergo cancer genetic testing (*p* < 0.001).

Specifically, WTP among patients (*n* = 354) was further assessed for potential associated factors. Table 3 shows the analysis in which only the level of education and income were found to be significantly associated with WTP. Patients with a higher level of education were more willing to pay for cancer genetic testing than patients with lower education levels. Patients with monthly household incomes of more than USD 1208.88 (MYR 5000) showed a higher percentage of WTP than those with an income of less than USD 1208.88 (MYR 5000).

### 3.3. Factors Influencing Willingness to Pay (WTP) for Cancer Genetic Testing Using Multivariate Logistic Analysis

Based on the bivariate analysis results, all factors with values of *p* < 0.25 were selected and further analyzed with multiple logistic regression analysis for willingness to pay using the enter method [21]. Table 4 shows the influencing factors of WTP for genetic testing. In general, patients with a low level of education and who were not willing to undergo cancer genetic testing had a decreased WTP for cancer genetic testing. If only among patients, having a low education level would decrease their WTP for cancer genetic testing. However, male patients had a decreased WTP for cancer genetic testing.

## 4. Discussion

With the increasing availability of genetic services, genetic testing programs’ counselling is necessary considering consumers’ interest in cancer risk information, which impacts various decision-making processes. Decisions about receiving cancer genetic testing services, including attending a genetics consultation and willingness to undergo testing, will depend on the individual’s knowledge, perceived risk of developing cancer, and attitude towards human genetics and its development [22]. A great part of the respondents in this study was willing to undergo cancer predisposition screening, similar to a previous study in Canada [23]. However, this high level of interest in undergoing screening may not always translate to real uptake of cancer genetic testing [23].

In another study done as a part of this research, family members of patients were found to be more willing to pay for cancer genetic testing [24]. The participants with a stronger family history of cancer may have an increased risk due to the family burden of disease, and finding out the results of the test may reduce the worry of not knowing whether they carry the faulty genes. However, those who are willing to pay for testing may not actually turn up to take the test. Studies of first-degree relatives of breast cancer patients demonstrated that while over 80% of the study participants gave affirmative answers to questions on wanting to be tested, only 50% gave blood samples for testing [25]. These individuals are an important group of patients of whom healthcare professionals should be aware and should refer for hereditary risk assessment [14]. Studies have found that genetic testing presents a dilemma about when and how to disclose genetic test results to relatives, which is critical for their acceptance of the fact that the earlier the cancer is found and treated, the better the results are [1,2,10,15]. Finding the “right” time and managing the time and content of disclosure can benefit the recipient and the informant to help reduce the chances of low inclination towards undertaking these tests [2,17].

Interestingly, younger respondents in this study were generally more willing to pay for testing if payment was out-of-pocket, revealing an inverse relationship between age and willingness to pay. Younger generations are usually less fatalistic about cancer and found to be more enthusiastic about genetic counselling [26]. Thus, these results reflect shifting norms among younger versus older individuals regarding the acceptability of health care services’ cost-sharing practices.

Other important socio-demographic factors associated with WTP for genetic testing were gender, marital status, total monthly household income, and education level. From the literature, WTP generally rose with income [27,28,29,30,31]. Moreover, women are typically found to be more anxious about health than men [25]. Another study on WTP for breast cancer screening also reported that WTP was greater among women with family history than those without, but WTP was reduced among women with a low household income [23]. Such studies evaluating the uptake of genetic testing among high-risk individuals usually include mostly female respondents; thus, they offer only a one-sided view of the population who could benefit from risk assessment. However, the risk population for certain cancers is not the same; there may be a difference of characteristics [14]. Further studies are needed to examine how men and women differ in their perceptions of risk, interest in, and willingness-to-pay for genetic testing due to differences in the level of risks. Additionally, more focus is needed on marginalized groups and minority ethnic groups who suffer from financial constraints and may not be willing to contribute much for their preventive health [15].

Concerns about whether having insurance would cover the costs of testing also influence individuals’ WTP [32]. In this study, those who had private insurance were more willing to pay than those who did not. The cost of testing could be a significant barrier in some groups due to low wages and not having health insurance [33]. Adams et al. also found that individuals were unwilling or unable to pay themselves if testing was expensive or not covered by their insurance [34]. The results in the present study indicate a reasonable level of interest in genetic testing but not much willingness to pay out-of-pocket. A reason could be that Malaysians are accustomed to a government-led health system where they generally do not pay much for medical health care services. This experience of relying on a public health care system may limit the extent to which respondents are willing to pay out-of-pocket for genetic tests as supported by Ries et al. [35]. Studies show that genetic assessment in high-risk individuals offers the greatest cancer prevention and cost-savings benefits when viewed from a societal perspective [35,36]; therefore, efforts are needed to understand how individuals value genetic testing and the barriers they may experience to undergo genetic testing in the setting of rising health care costs where out-of-pocket expenses are necessary.

In this study, WTP also correlated with the respondent’s education level, similar to previous studies [24,29,30,37]. This could be due to test-related concerns, which could be higher in less educated patients [38]. Many studies, including among Chinese, Australian, Hispanic, Poland, and African American samples, stated that most participants had insufficient knowledge about the importance of genetics in cancer and genetic testing [15,18]. In such scenarios, unsurprisingly, there would be an unwillingness to pay for the genetic testing for cancer risk. A study [25] reported that the respondents were willing to pay for the genetic testing based on their increased knowledge of the physical, emotional, and financial challenges associated with cancer management. Thus, a greater proportion of respondents were willing to pay for cancer genetic testing in order to determine their risk of developing cancer. Another researcher found that single individuals were more willing to pay, similar to a study where most of the respondents between 18 and 24 years old stated that reproductive decision making would have a moderate to strong effect on their interest in undergoing genetic testing [35]. This insight could be clinically useful to genetic service providers since reluctance to pursue testing among high-risk individuals may be rooted in specific barriers that, if more thoroughly explored, may lead to improved shared decision making. Despite its potential benefits, genetic research is a very sensitive topic for researchers, patients, the public, and ethicists alike [39]. Interestingly, most of the respondents in this study did not have such a moral objection. As a multicultural country, ethnicity and religion of the respondents were not a predictive factor for their willingness to undergo or pay for genetic testing. Instead, most of them were not opposed to the research in general.

Willingness to undergo genetic cancer screening appeared to be the most influential factor affecting WTP for it. This finding is similar to a study by Matro et al. [14] where the authors found that individuals with more positive attitudes towards genetic testing and cancer screening were more willing to pay for these services. On the other hand, fear of discovering a predisposition to a disease, disclosure of test results to insurers, and receiving the test from a specialist could be some factors inversely associated with willingness to undergo the genetic test [40]. Moreover, individuals who are not willing to undergo testing may have concerns about information overload, privacy, unclear benefits of the test, test accuracy, and testing procedures [5]. Thus, it is important to carefully consider the language used to convey genetic concepts and benefits of genomics studies to patients and their relatives. A key area for future research would be to understand how the language and presentation of genetic concepts affect patients’ willingness to undergo testing.

This study had a few limitations. As the research was conducted at health care facilities, all subjects were seeking health care services. Hence, this study could overestimate the uptake rates. Secondly, consumer surveys only report intentions based on what individuals perceive they would do. Qualitative research methods could be designed to understand more about the thought processes and motivation of respondents. Thirdly, cancer risk perception can influence the population’s interest in and WTP for cancer screening. Indeed, several prior studies on cancer susceptibility testing revealed that the higher the cancer risk perceptions are, the higher the level of interest in screening testing is [23]. This area also should be explored further in future research.

## 5. Conclusions

This pioneering study in Malaysia addressed the Malaysian population’s willingness to undergo and pay for cancer genetic testing. Overall, this suggests that in Malaysia, cancer patients and their relatives have mitigated enthusiasm towards cancer genetic testing if they have to pay out-of-pocket for it. With psychoeducation, the population would be more willing to undergo and pay for cancer genetic testing. This will increase the utilization of the offered services on genetics testing, and with cost-sharing practices with the insurance provider, they can be implemented population-wide. It is recommended that together with a cost-effectiveness study [41], this information could facilitate policymakers in designing and implementing a cancer screening program in future, whether offering risk rating on an individual basis or population-wide.

## Figures and Tables

**Table 1:** Willingness to pay for cancer genetic test (*n* = 641).

**Willingness to Pay (WTP)**	Frequency, *n*	Percentage (%)
**Payment Out-of-Pocket**		
Yes	98	15.3
No	208	32.4
Do not Know	335	52.3
**Percentage for co-payment**		
Yes	143	22.3
No	183	28.5
Do not Know	315	49.2
**Fully paid by government**		
Yes	291	45.4
No	64	10.0
Do not Know	286	44.6
**Fully paid by insurance**		
Yes	265 (41.4)	41.4
No	83 (12.9)	12.9
Do not Know	293 (45.7)	45.7

**Table 2:** Factors associated with WTP for cancer genetic testing in general (*n* = 641). Pearson’s chi-squared test was performed, level of significance set at *p* < 0.05.

Variable	WTP	Not WTP	Chi-Square	*p*-Value
	*n* (%)	*n* (%)		
**Family History of Cancer**				
Yes	33 (11.0%)	268 (89.0%)	8.197	0.004
No	65 (19.1%)	275 (80.9%)		
**Age (years)**				
<40	38 (20.9%)	144 (79.1%)	6.873	0.032
40–60	43 (14.1%)	262 (85.9%)		
>60	17 (11.0%)	137 (89.0%)		
**Gender**				
Male	25 (12.2%)	180 (87.8%)	10.286	0.01
Female	73 (16.7%)	363 (83.3%)		
**Ethnicity**				
Malays	65 (14.4%)	385 (85.6%)	0.831	0.362
Non-Malays	33 (17.3%)	158 (82.7%)		
**Religion**				
Muslim	66 (14.6%)	387 (85.4%)	0.617	0.432
Others	32 (17.0%)	156 (83.0%)		
**Level of education**				
Low	39 (08.9%)	401 (91.1%)	44.725	<0.001
High	59 (29.4%)	142 (70.6%)		
**Marital status**				
Married	72 (14.0%)	442 (86.0%)	3.286	0.070
Unmarried	26 (20.5%)	101 (79.5%)		
**Number of Children**				
Less than 3	51 (17.7%)	237 (82.3%)	2.364	0.124
3 or more	47 (13.3%)	306 (86.7%)		
**Income (MYR)**				
<5000	68 (12.9%)	461 (87.1%)	13.851	<0.001
≥5000	30 (26.8%)	82 (73.2%)		
**Insurance**				
Yes	32 (21.9%)	114 (78.1%)	6.415	0.011
No	66 (13.3%)	429 (86.7%)		
**Willingness to Undergo genetic testing**				
Yes	88 (23.3%)	289 (76.7%)	45.842	<0.001
No	10 (3.8%)	254 (96.2%)		

**Table 3:** Factors associated with WTP for cancer genetic testing among patients (*n* = 354).

Variable	WTP	Not WTP	Chi-Square	*p*-Value
	*n* (%)	*n* (%)		
**Family History of Cancer**				
Yes	18 (8.7%)	188 (91.3%)	1.105	0.293
No	18 (12.2%)	130 (87.8%)		
**Age (years)**				
<40	06 (12.8%)	41 (87.2%)	0.386 ^a^	0.824
40–60	19 (9.9%)	173 (90.1%)		
>60	11 (9.6%)	104 (90.4%)		
**Gender**				
Male	05 (5.4%)	87 (94.6%)	3.050	0.081
Female	31 (11.8%)	231 (88.2%)		
**Ethnicity**				
Malays	23 (9.7%)	214 (90.3%)	0.170	0.680
Non-Malays	13 (11.1%)	104 (88.9%)		
**Religion**				
Muslim	23 (9.7%)	215 (90.3%)	0.203	0.652
Others	13 (11.2%)	103 (88.8%)		
**Level of education**				
Low	19 (06.5%)	275 (93.5%)	26.092	<0.001
High	17 (28.3%)	43 (71.7%)		
**Marital status**				
Married	34 (10.9%)	279 (89.1%)	0.842 ^b^	0.359
Unmarried	02 (4.9%)	39 (95.1%)		
**Number of Children**				
Less than 3	15 (12.0%)	110 (88.0%)	0.709	0.400
3 or more	21 (9.2%)	208 (90.8%)		
**Income (MYR)**				
<5000	21 (07.0%)	277 (93.0%)	20.106	<0.001
≥5000	15 (26.8%)	41 (73.2%)		

Pearson’s chi-squared test was performed, level of significance set at *p* < 0.05; ^a^ likelihood ratio was used; ^b^ Yates correction was applied.

**Table 4:** Multiple logistic regression for factors influencing WTP for cancer genetic testing.

Variable	B	S.E.	Wald	*p*-Value	Adj. OR	95% C.I.
**General**						
Status (Patient)	−1.256	0.459	7.477	0.006	0.285	0.116–0.701
Level of education (Low)	−1.044	0.300	12.128	<0.001	0.352	0.196–0.633
Willingness to Undergo genetic test (No)	−2.142	0.372	33.116	<0.001	0.117	0.057–0.244
Constant	0.472	0.720	0.430	0.512	1.603	
**Patients**						
Level of education (Low)	1.331	0.421	9.974	0.002	3.783	1.657–8.640
Gender (Male)	−0.519	0.260	3.976	0.046	0.595	0.357–0.991
Constant	−0.566	0.331	2.931	0.087	0.568	

Nagelkerke’s R2 = 0.275, 0.126; Hosmer–Lemeshow test = *p*-value = 0.884, 0.638; percentage of correct classification = 85.3, 84.7.

## Data Availability

The data presented in this study are available within the article.
